# Long-Term Efficacy and Anticoagulation Strategy of Left Atrial Appendage Occlusion During Total Thoracoscopic Ablation of Atrial Fibrillation to Prevent Ischemic Stroke

**DOI:** 10.3389/fcvm.2022.853299

**Published:** 2022-03-31

**Authors:** Ju Youn Kim, Dong Seop Jeong, Seung-Jung Park, Kyoung-Min Park, June Soo Kim, Young Keun On

**Affiliations:** ^1^Division of Cardiology, Department of Internal Medicine, Samsung Medical Center, Heart Vascular and Stroke Institute, Sungkyunkwan University School of Medicine, Seoul, South Korea; ^2^Department of Thoracic and Cardiovascular Surgery, Samsung Medical Center, Sungkyunkwan University School of Medicine, Seoul, South Korea

**Keywords:** atrial fibrillation, appendage, thoracoscope surgery, anticoagulation, ischemic stroke

## Abstract

**Objectives:**

Atrial fibrillation (AF) is associated with an increased ischemic stroke, and the left atrial appendage (LAA) represents the main source of thrombus formation. We evaluated the long-term efficacy of surgical thoracoscopic LAA occlusion during total thoracoscopic ablation of AF to prevent the stroke and anticoagulation strategy after surgery.

**Methods:**

Patients who underwent total thoracoscopic ablation for AF, from February 2012 to May 2020, were included; Patients who did not receive LAA occlusion were excluded. We evaluated the development of thromboembolism in these patients.

**Results:**

The total number of 460 patients [mean age, 57.1 ± 9.2 years; 400 (87.0%) males] were included in the study. The mean follow-up duration was 44.8 months. The mean CHA_2_DS_2_-VASc score was 1.9 ± 1.6. Median OAC duration was 109.5 days after the surgery, and the final number of patients who discontinued OAC were 411 (89.3%) in total. Anticoagulation discontinuation rate according to CHA_2_DS_2_-VASc score are as follows; (i) 0 = 99.0%; (ii) 1 = 98.2%; and (iii) ≥2 = 81.3%. The annualized incidence rate of ischemic stroke was 0.78%/year, showing a 73% risk reduction compared with the CHA_2_DS_2_-VASc predicted rate without anticoagulation. The hazard ratio for ischemic stroke according to previous stroke history was 1.5 [95% confidential interval (CI) 0.3–7.3, *p* = 0.62], and that of remnant LAA was 5.1 (1.2–20.9, *p* = 0.02).

**Conclusions:**

Thoracoscopic LAA occlusion during total thoracoscopic ablation of AF was effective to prevent ischemic stroke. Most patients could discontinue OAC therapy after the procedure. Patients who had a residual trabeculated LAA, or peri-occluder pouch in follow-up CT need to maintain OAC therapy even after LAA occlusion.

## Introduction

Atrial fibrillation (AF) is associated with an increased risk of ischemic stroke (1). The etiology of ischemic stroke secondary to AF is cardio-embolism, and the most common site of thrombus formation is the left atrial appendage (LAA). Oral anticoagulants are effective on the prevention of thromboembolism. However, some patients experienced an ischemic stroke during the treatment of anticoagulation. Furthermore, anticoagulation increases the risk of bleeding and can cause life-threatening events. LAA closure with devices or surgical LAA occlusion is a potential alternative for this population (2–5). LAA occlusion using a closure device combined with catheter ablation for AF can be performed safely in a single procedure at a reduced stroke rate (6). However, device-related thrombus after LAA closure can develop and is associated with a higher rate of stroke (7). LAA occlusion during other cardiac surgery procedures also reduced stroke risk compared with the no-occlusion group (4). Thoracoscopic ablation is a less invasive approach without opening the cardiac chamber for stand-alone surgery for treatment of AF. Concomitant occlusion of the LAA through a thoracoscopic approach could be performed. The surgical occlusion device is placed epicardially to exclude the trabeculated LAA (8). Little is known about the long-term outcomes and feasibility of these procedures.

In this study, we evaluated the long-term efficacy of surgical thoracoscopic LAA occlusion during total thoracoscopic ablation of AF to prevent ischemic stroke and the anticoagulation strategy after surgery.

## Methods

### Study Population

**Figure 1:**
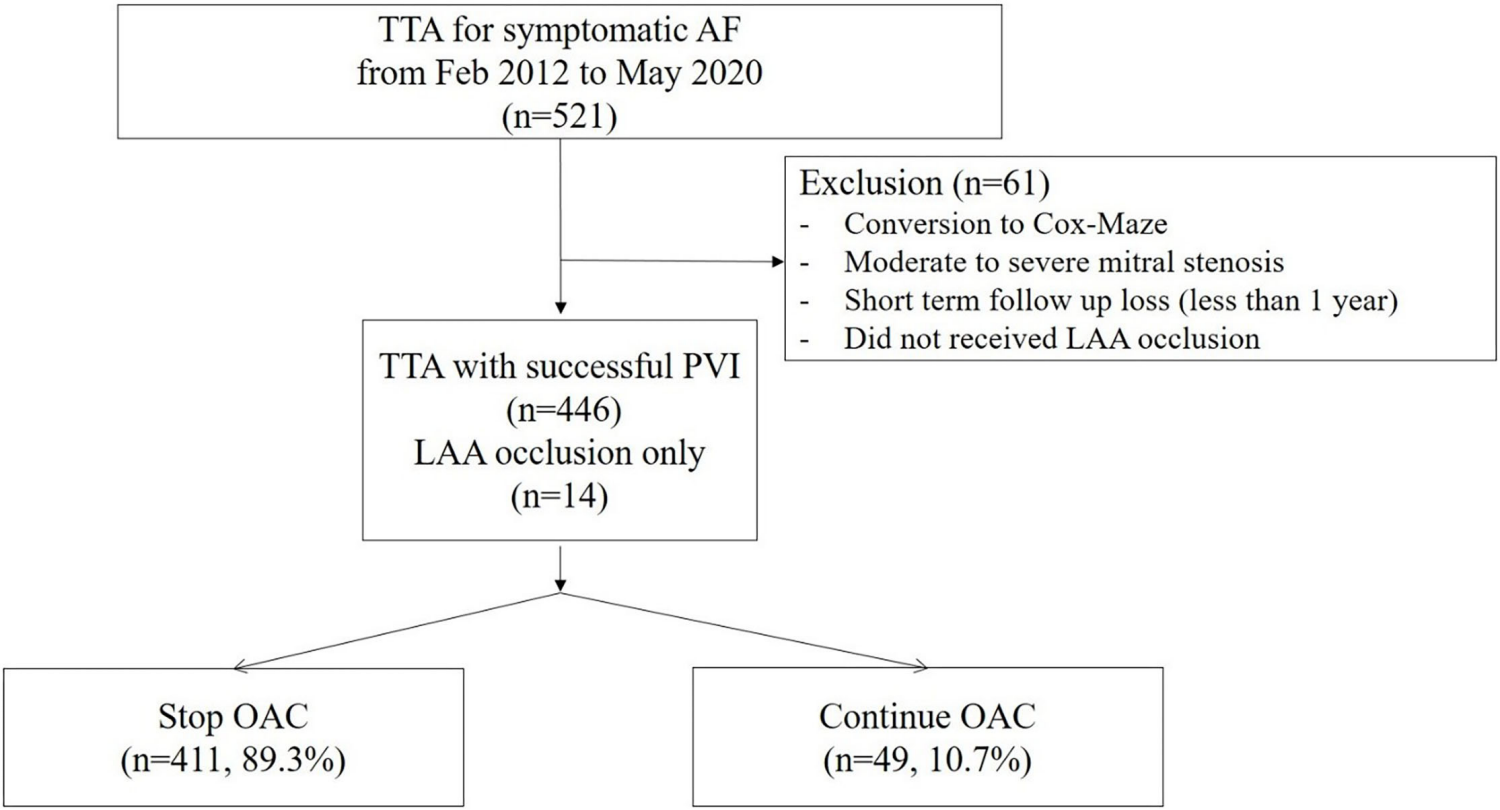
Patient flow chart.

This study was a single-center, retrospective, observational study. Consecutive patients who underwent total thoracoscopic ablation for AF, from February 2012 to May 2020, were included. Patients who converted to open Cox–Maze surgery exhibited underlying moderate to severe mitral stenosis or were subjected to short-term follow-up loss after <1 year were excluded. In addition, patients who did not receive LAA occlusion were excluded. Patients who underwent unilateral pulmonary vein isolation (PVI) or thoracoscopic LAA occlusion only without AF ablation were included (Figure 1). We evaluated the development of thromboembolism during follow-ups. This study was approved by the Institutional Review Board of Samsung Medical Center, South Korea (IRB No. 2020-06-159).

### Surgical Techniques

**Figure 2:**
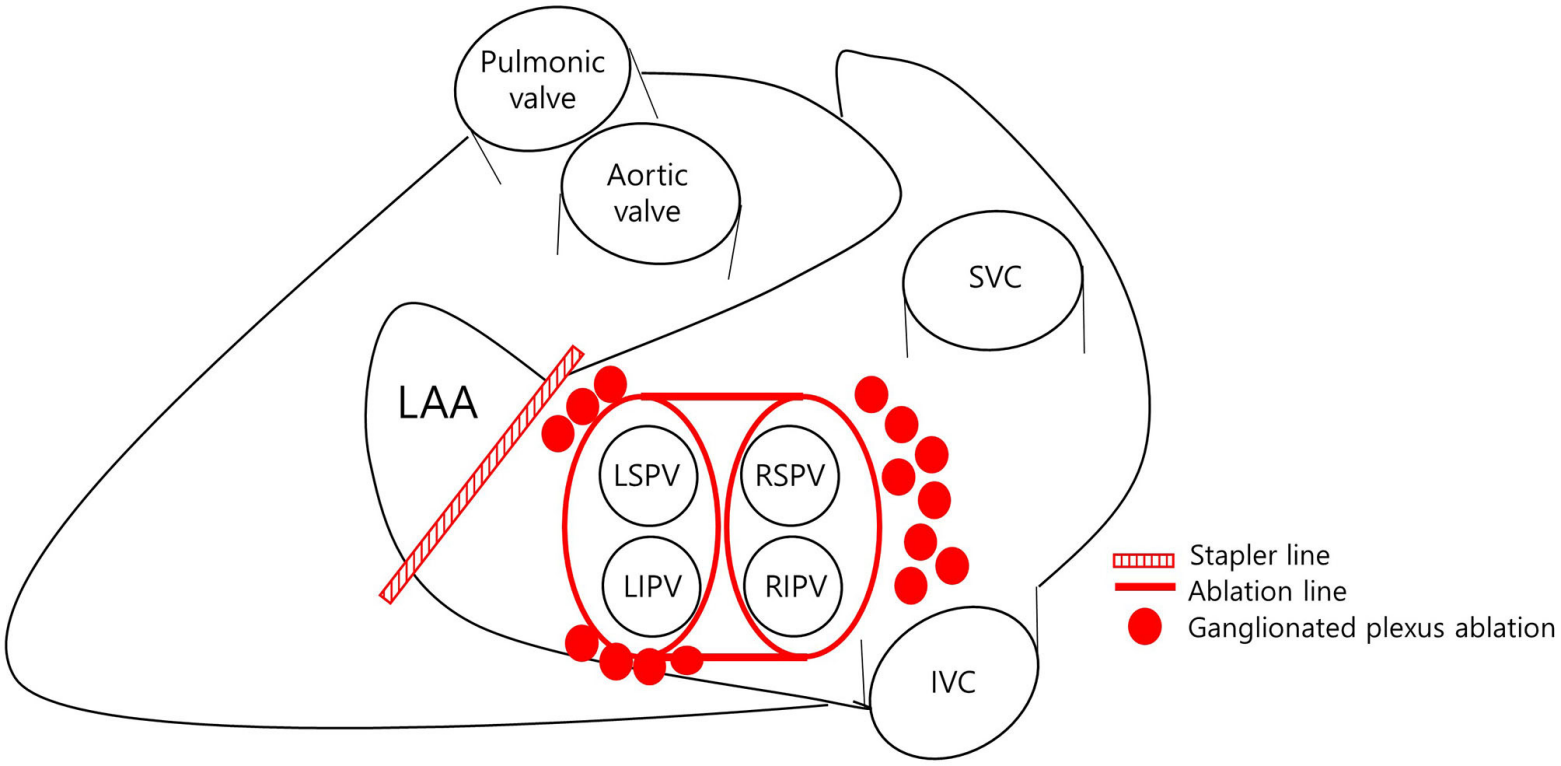
Ablation strategy. LAA, left atrial appendage; LSPV, left superior pulmonary vein; LIPV, left inferior pulmonary vein; RSPV, right superior pulmonary vein; RIPV, right inferior pulmonary vein; SVC, superior vena cava; IVC, inferior vena cava.

Before surgery, all patients underwent transesophageal echocardiography (TEE) to exclude LAA thrombus. Total thoracoscopic ablation procedures were performed under general anesthesia. All procedures were performed using standard techniques as described previously (9). The bilateral thoracoscopic approach was used with a video-assisted thoracoscopic surgical technique. Beginning on the right side, a 5-mm port was introduced into the fourth intercostal space at the mid-axillary line. After carbon dioxide insufflation to expand the operative field and depress the diaphragm, the remaining two ports were placed into the third intercostal space at the anterior axillary line and the sixth intercostal space at the mid-axillary line. After pericardial tenting, a lighted dissector (AtriCure Lumitip Dissector, Atricure, Inc., Cincinnati, OH, USA) was used to pass a rubber band under the PV antrum through the oblique sinus. An AtriCure Isolator Transpolar Clamp (Atricure, Inc.) was connected to the rubber band and positioned around the PV antrum. PV antrum isolation was performed by applying bipolar radiofrequency energy 6 times to the clamps around the PV antrum. Additional superior and inferior ablation lines connecting both PV isolation lines were created epicardially using a linear pen device (MLP, Atricure, Inc.). Ganglionated plexi subsequently were ablated with bipolar radiofrequency energy with the aid of high-frequency stimulation. Confirmation of ablation lines was obtained by pacing testing using the AtriCure Cooltip pen (MAX5, Atricure). The procedure was repeated on the left side. Before PV and ganglionated plexus ablation, the ligament of Marshall was dissected and ablated. When all ablations were complete and the conduction block was confirmed, the left atrial appendage was removed using an Echelon Flex 60 articulating endoscopic linear stapler (Ethicon Endo-Surgery Inc., Cincinnati, OH, USA) (Figure 2).

### Outcome

The primary outcome was the occurrence of ischemic stroke and thromboembolism after surgery. The etiology of ischemic stroke was evaluated and determined to be procedure-related, cardio-embolic, or resulted from small artery occlusion.

### Follow-Up

All patients were followed up by 2 weeks, 3 months, 6 months, and every 6 months thereafter.

Electrocardiography (ECG) was performed at each visit, and 24-h Holter monitoring was performed at three, six, and 12 months and annually thereafter. Additional monitoring was performed when patients experienced tachyarrhythmia symptoms. Follow-up computed tomography (CT) –angiography was performed to identify residual LAA or LA thrombus formation at least 6 months afterwards. Successful LAA occlusion was defined as the absence of a residual trabeculated LAA stump. Other findings such as LA thrombus, accessory appendage, and remnant peri-occluder pouch were evaluated. Oral anticoagulant (OAC) was resumed 1 or 2 days after the procedure with complete hemostasis and continued for at least 3 months.

### Statistical Analysis

Statistical analysis was performed using SPSS ver. 27.0 software (SPSS Inc., Chicago, IL, USA). Continuous variables were compared using the unpaired *t*-test or Wilcoxon rank-sum test, and categorical variables were compared using either the Chi-squared test or Fisher's exact test as determined appropriate. The incidence rates of clinical events are presented as person-years and events rate curves were obtained by Kaplan–Meier analysis. The risk of thromboembolism was assessed using a Cox proportional hazards model, and is presented as the hazard ratio (HR). A *p*-value < 0.05 was considered significant.

## Results

### Baseline Characteristics

**Figure 3:**
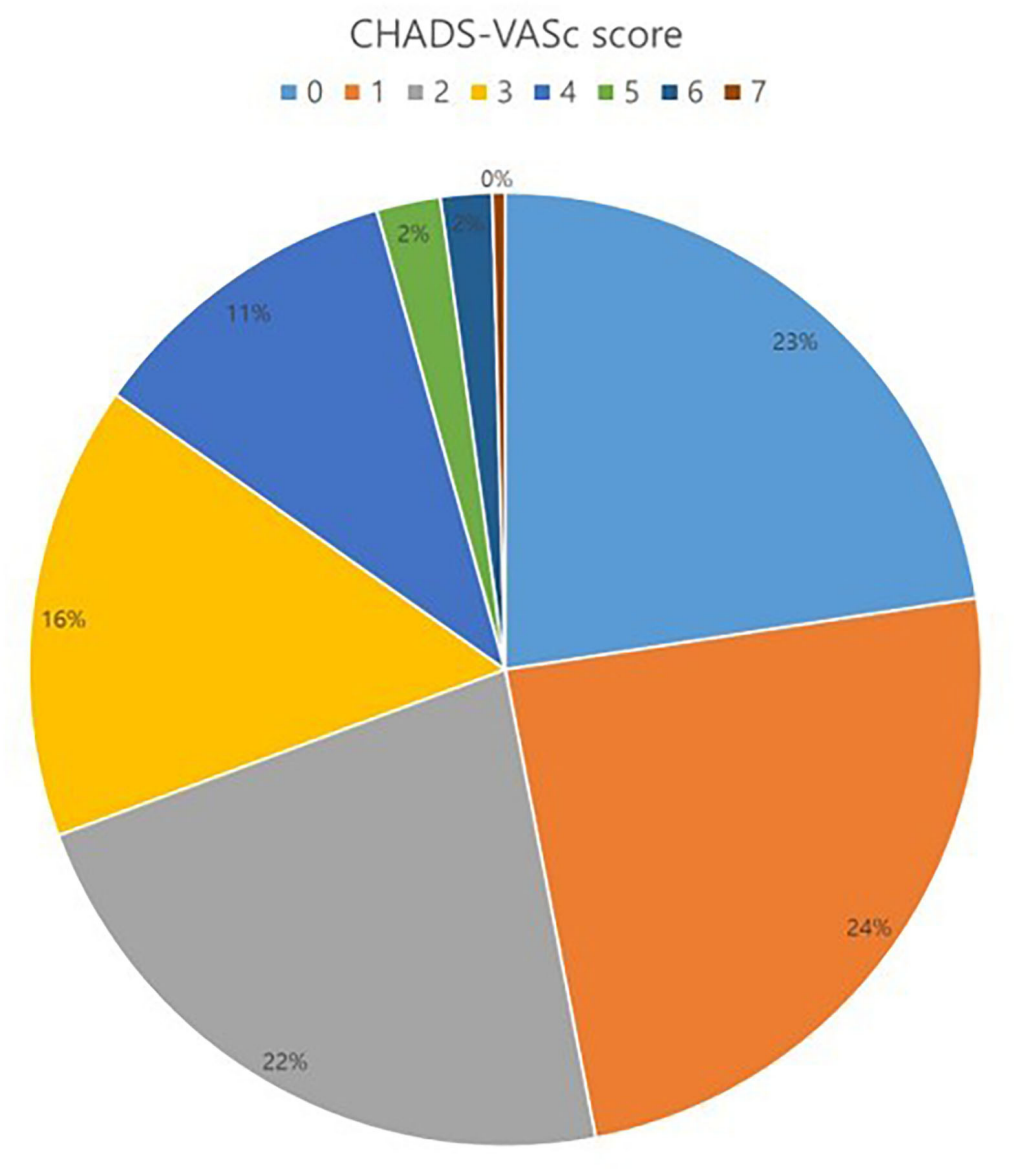
Distribution of CHA_2_DS_2_-VASc score.

The total number of 521 patients underwent the total thoracoscopic ablation procedure, and 61 were excluded from the study. Fifteen patients were converted to open Cox–Maze surgery, three patients were mitral stenosis, 16 patients were lost to follow-up after <1 year, and 27 patients did not receive LAA occlusion due to advanced heart failure or small LAA. 460 patients [mean age, 57.1 ± 9.2 years; 400 (87.0%) males] were included for analysis. Among these patients, 14 received only thoracoscopic LAA occlusion without AF ablation. The mean follow-up duration was 44.8 months. 385 (83.7%) patients exhibited persistent AF and 94 (20.4%) had a previous history of ischemic stroke. The median OAC duration was 109.5 days after the surgery, and the total of 411 (89.3%) patients discontinued OAC. Anticoagulation discontinuation rate according to CHA_2_DS_2_-VASc score are as follows; (i) 0 = 99.0%; (ii) 1 = 98.2%; and (iii) ≥2 = 81.3%. The mean CHA_2_DS_2_-VASc score was 1.9 ± 1.6, and 104 (22.6%) patients exhibited score 0 (Figure 3). The mean CHA_2_DS_2_-VASc score excluding the score 0 was 2.4 ± 1.3. Follow-up CT was performed in 337 (73.3%) patients, and 26 (7.7%) patients exhibited remnant LAA. Total 277 (60.2%) patients have maintained sinus rhythm during overall follow-up. The one- and two-year atrial tachyarrhythmia–free survival rates were 84.5 and 70.9%, respectively. The annualized recurrence rate of atrial tachyarrhythmia was 14.2%/year. The other baseline characteristics are summarized in Tables 1, 2.

**Table 1:** Baseline characteristics of overall population.

**Variables**	**All patients (*N* = 460)**
Age (years)	57.1 ± 9.2
Sex (male) (*n*, %)	400 (87.0%)
Hypertension (*n*, %)	230 (50.0%)
Diabetes mellitus (*n*, %)	60 (13.0%)
Previous stroke (*n*, %)	94 (20.4%)
Congestive heart failure (*n*,%)	73 (15.9%)
CHA_2_DS_2_-VASc	1.9 ± 1.6
AF type (*n*, %)
Paroxysmal	75 (16.3%)
Persistent	100 (21.7%)
LS persistent	285 (62.0%)
EF (%)	59.7 ± 7.2
LA diameter (mm)	46.3 ± 7.2
LA volume index (ml/m^2^)	49.7 ± 17.3
E/e‘	8.91 ± 3.54

*AF, atrial fibrillation; EF, ejection fraction; LA, left atrium; LS, long standing*.

**Table 2:** Baseline characteristics between patients with or without stroke.

**Variables**	**Stroke patients (*N* = 13)**	**Non-stroke patients** **(*N* = 447)**	***P*-value**
Age (years)	62.8 ± 9.4	57.0 ± 9.1	0.05
Sex (male) (*n*, %)	9 (69.2%)	391 (87.5%)	0.08
Hypertension (*n*, %)	6 (46.2%)	224 (50.1%)	1.00
Diabetes mellitus (*n*, %)	3 (23.1%)	57 (12.8%)	0.39
Previous stroke (*n*, %)	4 (30.8%)	90 (20.1%)	0.48
Congestive heart failure (*n*, %)	4 (30.8%)	69 (15.4%)	0.24
CHA_2_DS_2_-VASc	4.5 ± 1.7	1.8 ± 1.5	<0.001
AF type (*n*, %)			0.56
Paroxysmal	1 (7.7%)	74 (16.6%)	
Persistent	2 (15.4%)	98 (21.9%)	
LS persistent	10 (76.9%)	275 (61.5%)	
EF (%)	58.7 ± 6.2	59.7 ± 7.3	0.62
LA diameter (mm)	45.8 ± 10.2	46.3 ± 7.1	0.78
LA volume index (ml/m^2^)	56.1 ± 31.7	49.5 ± 16.7	0.19
E/e‘	11.5 ± 5.6	7.5 ±3.7	0.25
OAC treatment	4 (30.8%)	45 (10.1%)	0.04
Incomplete LAA occlusion	3 (33.3%)	23 (7.0%)	0.03
AF recurrence	7 (53.8%)	176 (39.4%)	0.39

*AF, atrial fibrillation; EF, ejection fraction; LA, left atrium; LS, long standing; OAC, oral anticoagulation*.

### Outcomes

**Figure 4:**
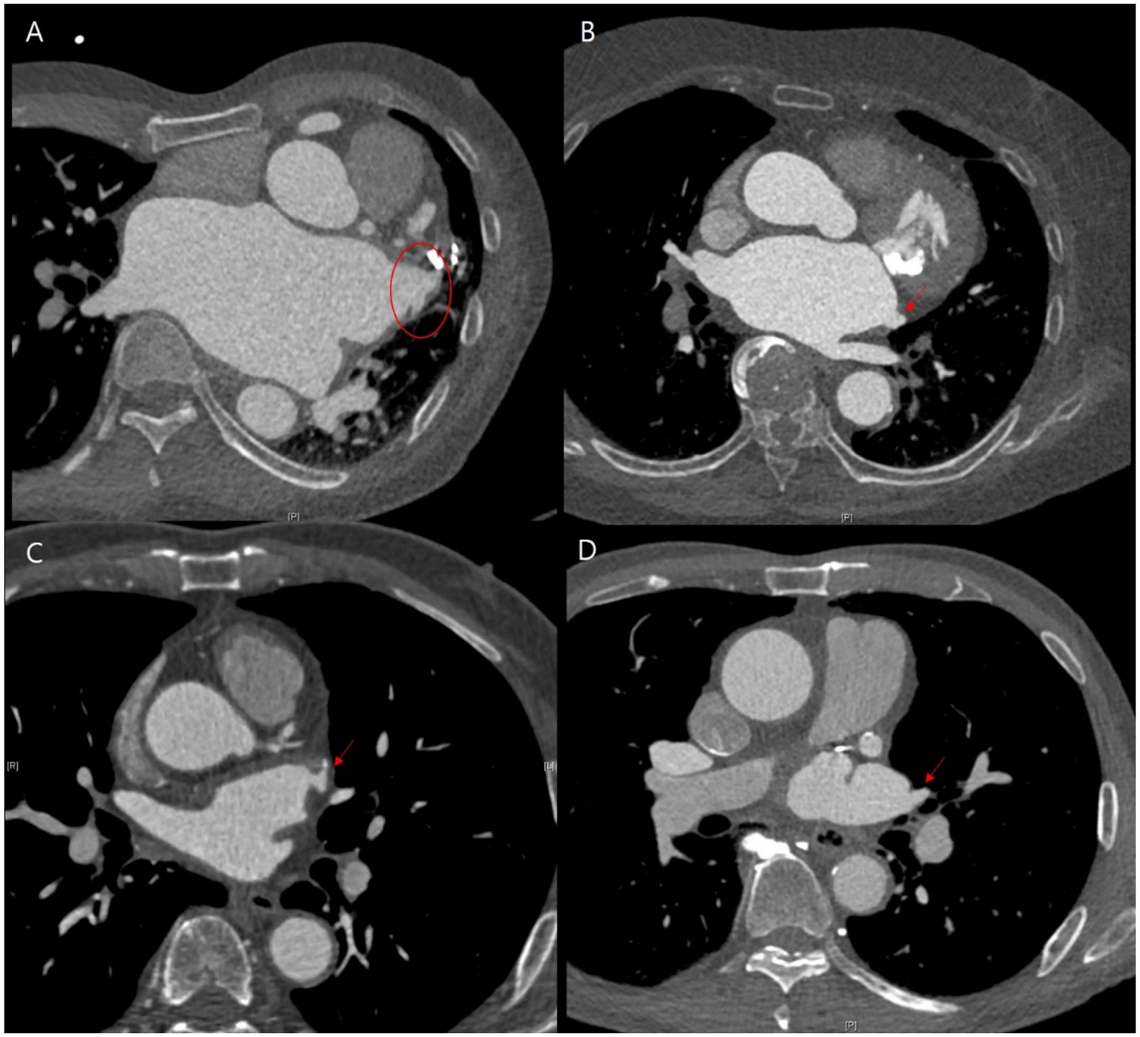
Representative cases of follow-up computed tomography (CT) findings that showed residual left atrial appendage (LAA). **(A,B)** Patients exhibited the occurrence of ischemic stroke, while **(C,D)** did not. **(A)** Residual trabeculated LAA. **(B)** Left atrial accessory appendage and mitral annular calcification without functional mitral stenosis. **(C,D)** Remnant LAA pouch.

**Figure 5:**
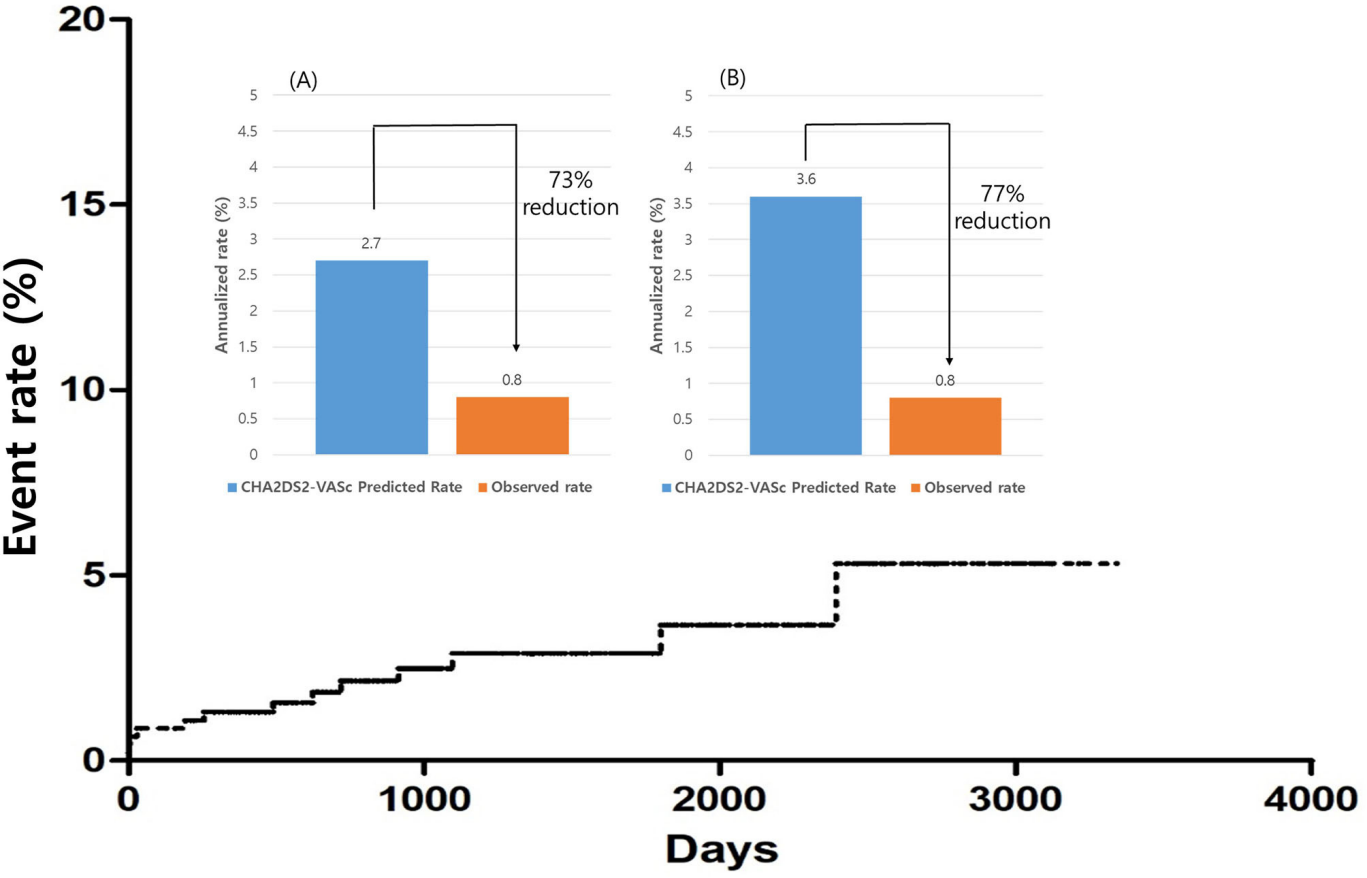
Overall ischemic stroke event rates. Relative ischemic stroke reduction compared with the predicted rate. **(A)** Overall patients. **(B)** Patients other than that with CHA_2_DS_2_-VASc score 0.

The annualized incidence rate of ischemic stroke was 0.78%/year, comprising 13 patients, which demonstrated a 73% risk reduction compared with the CHA_2_DS_2_-VASc-predicted rate without anticoagulation (10). Four patients exhibited procedure related ischemic stroke – three occurred <4 days after the surgery and one was stroke related with an atrial-esophageal fistula after 1 month of the surgery. Four patients had a history of previous embolic stroke. Only one patient experienced an ischemic stroke during OAC therapy. That patient had a history of three previous embolic strokes, and follow-up CT revealed remnant LAA (Figure 4). Patient characteristics are summarized in Table 3. The annualized incidence rate of ischemic stroke excluding the CHA_2_DS_2_-VASc score 0 was 0.84%/year, showing a 77% risk reduction (Figure 5). Hospitalization due to heart failure aggravation was observed in two patients during follow-up. The procedure related complications are summarized in Table 4.

**Table 3:** Detailed characteristics of patients who developed ischemic stroke.

**Cases**	**CHA_**2**_DS_**2**_-VASc score**	**Previous stroke history**	**Major stroke (1)** **TIA (2)**	**Procedure related stroke**	**OAC treatment**	**Residual LAA in CT**	**AF recurrence**
1	3	0	1	1	0		1
2	3	0	1	0	0		1
3	0	0	1	1	0	0	1
4	4	1	1	1	0		1
5	4	0	2	0	0	0	0
6	4	0	1	0	0	0	0
7	1	0	1	0	0	1	0
8	4	1	2	0	0	0	0
9	0	0	2	0	0		1
10	1	0	1	0	0	0	0
11	4	0	1	0	0	1	0
12	4	1	1	1	0	0	1
13	4	1	1	0	1	1	1

*TIA, transient ischemic attack; OAC, oral anticoagulant; LAA, left atrial appendage; CT, computed tomography; AF, atrial fibrillation*.

**Table 4:** Procedure-related complications.

	**All patients (*n* = 460)**
Total	25 (5.4%)
Pacemaker insertion due to bradycardia	5 (1.1%)
Atrioesophageal fistula	1 (0.2%)
Pericarditis	16 (3.5%)
Pleuritis	3 (0.7%)

**Figure 6:**
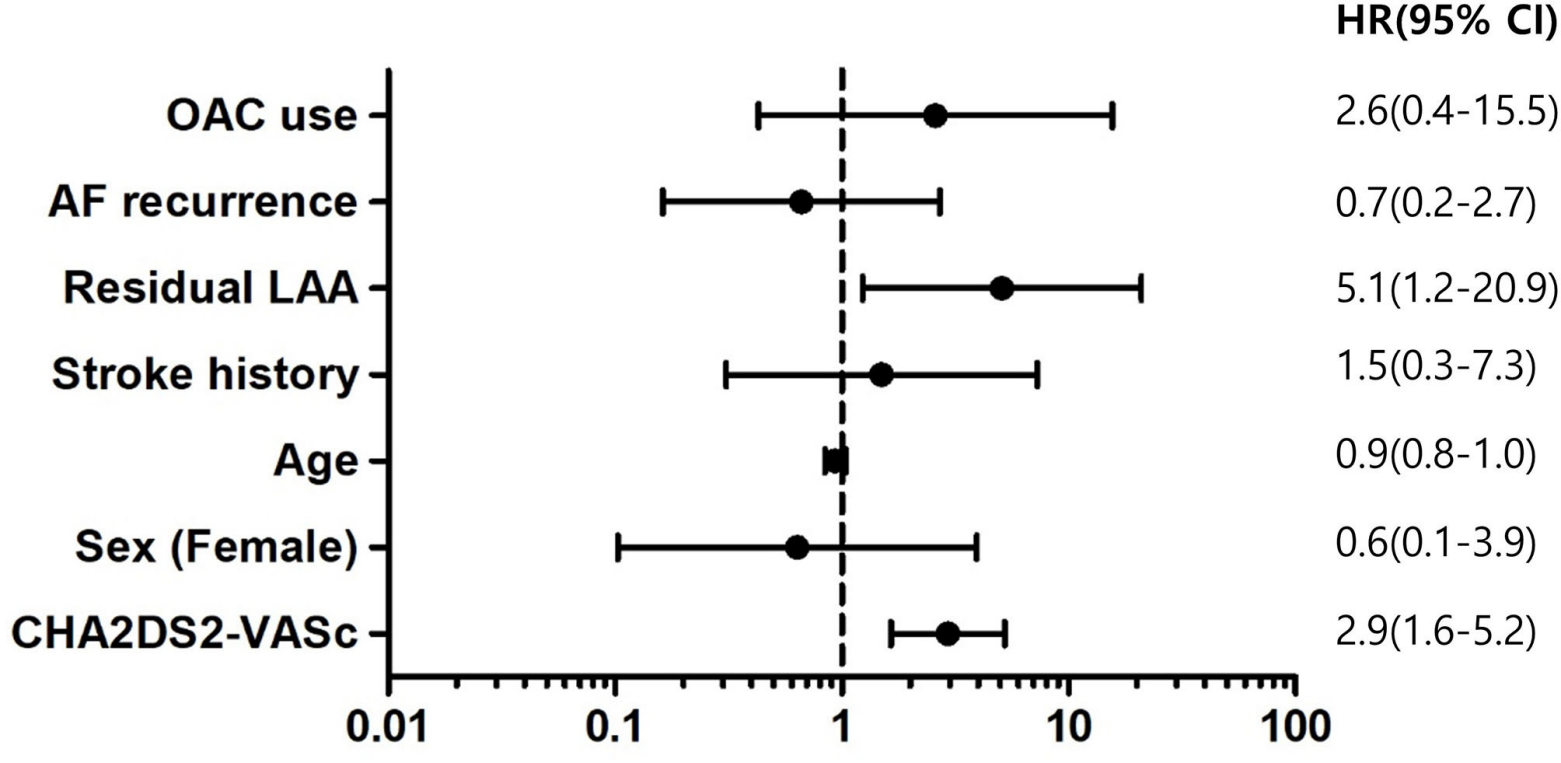
Hazard ratios for ischemic stroke.

The hazard ratio for ischemic stroke according to previous stroke history was 1.5 [95% confidential interval (CI) 0.3–7.3, *p* = 0.62], those of remnant LAA was 5.1 (1.2–20.9, *p* = 0.02), and CHA_2_DS_2_-VASc was 2.9 (1.6–−5.2, *p* < 0.001). The recurrence of AF or the use of OAC was not associated with ischemic stroke. The hazard ratio of the recurrence of AF was 0.7 (0.2–2.7, *p* = 0.57), that of OAC use was 2.6 (0.4–15.5, *p* = 0.30) and age was 0.9 (0.8–1.0, *p* = 0.21) (Figure 6).

## Discussion

This is the largest and longest follow-up study evaluating the efficacy of thoracoscopic LAA occlusion during lone AF surgery for ischemic stroke prevention. Thoracoscopic LAA occlusion can prevent ischemic stroke with a 73% risk reduction. Patients who had previous stroke history or residual LAA in CT findings should maintain OAC therapy even after LAA occlusion.

### The Effect of LAA Occlusion

LAA represents the sources of thrombus formation in patients with AF associated with blood stasis. Decreased LAA peak flow velocity is associated with increased thromboembolic risk (11). In addition, LAA morphology correlates with the risk of stroke, especially with an increased number of lobes independent of blood stasis (12). In this regard, several methods were used to perform occlusion of LAA including surgical resection or LAA device occlusion. LAA occlusion with a device can be considered in patients with higher stroke or bleeding risk. The ischemic stroke rate was reduced by 67% with device closure. However, device-related thrombosis was seen in 1.6% of patients (13). Recently, LAA occlusion during cardiac surgery among patients accompanied with AF exhibited a benefit for ischemic stroke prevention compared with the non-occlusion group (4). Several methods including amputation and closure, stapler closure, double-layer linear closure, or closure with a surgical occlusion device were performed without complications or increase in the risk of heart failure or major bleeding. The study included patients scheduled to undergo cardiac surgery with cardiopulmonary bypass and excluded those who underwent off-pump surgery. Cardiopulmonary bypass surgery itself has the risk of thrombus formation compared with off-pump surgery (14). Therefore, early events during the first 30 days after the surgery showed no difference between the two groups associated with peri-procedural stroke. In addition, concomitant surgical ablation of AF was performed in about 30% of patients in both groups in the study. Another small study demonstrated that thoracoscopic ablation with appendage ligation could prevent recurrent stroke in patients with AF with a previous stroke compared to medical therapy (15). In our study, we conducted minimally invasive surgery using video-assisted thoracoscopy concomitant with AF rhythm control surgery without cardiopulmonary bypass. 94 (20.4%) patients had a previous history of ischemic stroke. Although, peri-procedural stroke was observed only in 0.8% of patients. Furthermore, 97% of patients underwent surgical ablation of AF. Maintaining sinus rhythms can have an influence on the risk reduction of thrombus formation after surgery.

The function of the LAA is not well-known. In animal studies, removal of the LAA resulted in decreased compliance of the LA, which was associated with decreased reservoir function (16). In our study, only two exhibited aggravation of heart failure during follow-up. We conducted LAA occlusion using an articulating endoscopic linear stapler targeting the removal of the trabeculated portion and preservation of the basal portion of the LAA, which was confirmed with CT angiography after surgery. This might have affected LA function maintenance with lowered risk of thrombus formation.

### Anticoagulation Strategy

Most of the patients discontinued OAC during the follow-up, and the median OAC use-duration was 109.5 days in our study. Nearly 90% of patients discontinued OAC but showed 73% risk reduction compared with the CHA_2_DS_2_-VASc predicted rate without anticoagulation. Very few patients developed ischemic stroke after OAC discontinuation.

Lee et al. reported that LAA ligation or stapled excision may increase the embolic risk compared to surgical excision technique due to incompletely elimination of LAA (17). Our study showed that the remnant trabeculated LAA or peri-occluder pouch confirmed with CT was associated with increased ischemic stroke risk. The previous history of stroke exhibited a tendency to increase the risk of ischemic stroke. Therefore, CT findings after surgery were important in determining whether to continue anticoagulation therapy. In addition, patients who had a history of recurrent ischemic stroke should consider continuing OAC therapy.

## Limitations

This is a single-center, single-arm, retrospective registry cohort study. However, most of our patients received a standardized strategy and the same follow-up protocol. Although there was no control group of non-occlusion, the effectiveness of ischemic stroke prevention has been demonstrated through comparisons with CHA_2_DS_2_-VASc predicted rates. The major limitation of this study is that the mean CHA_2_DS_2_-VASc score included in the study was 1.9. Patients with CHA_2_DS_2_-VASc 0 scores have low ischemic stroke rates, and current guidelines recommend no stroke prevention treatment in this group (18). Also, 22% of patients had a CHA_2_DS_2_-VASc score of 0, hindering discussion of the efficacy of LAA occlusion for these patients. Therefore, we analyzed patients who had CHA_2_DS_2_-VASc scores of, at least, 1 point and showed greater risk reduction in preventing stroke in this group. In our study, most of the patients had lone AF and our cohort consisted of relatively younger patients. Age is an important risk factor in ischemic stroke, so older patients need to be studied further. Lastly, we included patients who underwent unilateral pulmonary vein isolation or thoracoscopic LAA occlusion only. Rhythm status may affect the occurrence of ischemic stroke, however, the main purpose of this study was to identify whether LAA occlusion is effective in preventing ischemic stroke. Our study results suggested that residual LAA was a risk factor for ischemic stroke.

## Conclusion

Thoracoscopic LAA occlusion during total thoracoscopic ablation of AF was effective in preventing ischemic stroke without any increase of the additional complications or development of heart failure. Most patients could discontinue OAC therapy after the procedure. Patients who had a residual trabeculated LAA or peri-occluder pouch in follow-up CT need to maintain OAC therapy even after LAA occlusion.

## Data Availability Statement

The original contributions presented in the study are included in the article/supplementary material, further inquiries can be directed to the corresponding author.

## Ethics Statement

The studies involving human participants were reviewed and approved by Institutional Review Board of Samsung Medical Center, South Korea (IRB No. 2020-06-159). Written informed consent for participation was not required for this study in accordance with the national legislation and the institutional requirements.

## Author Contributions

JYK contributed to study design, data analysis, data interpretation, and writing of the report. DJ contributed to data acquisition, writing, and critical revision of the report. S-JP, K-MP, and JSK contributed to critical revision of the report. YO contributed to study conception and design, data interpretation, and critical revision of the report. All authors contributed to the article and approved the submitted version.

## Conflict of Interest

The authors declare that the research was conducted in the absence of any commercial or financial relationships that could be construed as a potential conflict of interest.

## Publisher's Note

All claims expressed in this article are solely those of the authors and do not necessarily represent those of their affiliated organizations, or those of the publisher, the editors and the reviewers. Any product that may be evaluated in this article, or claim that may be made by its manufacturer, is not guaranteed or endorsed by the publisher.

## References

[1] Wolf PA Abbott RD Kannel WB. Atrial fibrillation as an independent risk factor for stroke: the Framingham Study. Stroke. (1991) 22:983–8. 10.1161/01.STR.22.8.983 1866765

[2] Yao X Gersh BJ Holmes DR Melduni RM Johnsrud DO Sangaralingham LR. Association of surgical left atrial appendage occlusion with subsequent stroke and mortality among patients undergoing cardiac surgery. JAMA. (2018) 319:2116–26. 10.1001/jama.2018.6024 29800182 PMC6351069

[3] Holmes DR Reddy VY Gordon NT Delurgio D Doshi SK Desai AJ. Long-term safety and efficacy in continued access left atrial appendage closure registries. J Am Coll Cardiol. (2019) 74:2878–89. 10.1016/j.jacc.2019.09.064 31806131

[4] Whitlock RP Belley-Cote EP Paparella D Healey JS Brady K Sharma M. Left atrial appendage occlusion during cardiac surgery to prevent stroke. N Engl J Med. (2021) 384:2081–91. 10.1056/NEJMoa2101897 33999547

[5] Yoshimoto A Suematsu Y Kurahashi K Kaneko H Arima D Nishi S. Early and middle-term results and anticoagulation strategy after left atrial appendage exclusion using an epicardial clip device. Ann Thorac Cardiovasc Surg. (2021) 27:185–90. 10.5761/atcs.oa.20-00204 33208590 PMC8343034

[6] Alipour A Swaans MJ van Dijk VF Balt JC Post MC Bosschaert MAR. Ablation for atrial fibrillation combined with left atrial appendage closure. JACC Clin Electrophysiol. (2015) 1:486–95. 10.1016/j.jacep.2015.07.009 29759402

[7] Dukkipati SR Kar S Holmes DR Doshi SK Swarup V Gibson DN. Device-related thrombus after left atrial appendage closure: incidence, predictors, and outcomes. Circulation. (2018) 138:874–85. 10.1161/CIRCULATIONAHA.118.035090 29752398

[8] Ailawadi G Gerdisch MW Harvey RL Hooker RL Damiano RJ Salamon T. Exclusion of the left atrial appendage with a novel device: early results of a multicenter trial. J Thorac Cardiovasc Surg. (2011) 142:1002–9. 10.1016/j.jtcvs.2011.07.052 21906756

[9] On YK Park K-M Jeong DS Park PW Lee YT Park S-J. Electrophysiologic results after thoracoscopic ablation for chronic atrial fibrillation. Ann Thorac Surg. (2015) 100:1595–603. 10.1016/j.athoracsur.2015.04.127 26215779

[10] Friberg L Rosenqvist M Lip GYH. Evaluation of risk stratification schemes for ischaemic stroke and bleeding in 182 678 patients with atrial fibrillation: the Swedish atrial fibrillation cohort study. Eur Heart J. (2012) 33:1500–10. 10.1093/eurheartj/ehr488 22246443

[11] Zabalgoitia M Halperin JL Pearce LA Blackshear JL Asinger RW Hart RG. Transesophageal echocardiographic correlates of clinical risk of thromboembolism in nonvalvular atrial fibrillation. stroke prevention in atrial fibrillation III Investigators. J Am Coll Cardiol. (1998) 31:1622–6. 10.1016/S0735-1097(98)00146-6 9626843

[12] Di Biase L Santangeli P Anselmino M Mohanty P Salvetti I Gili S. Does the left atrial appendage morphology correlate with the risk of stroke in patients with atrial fibrillation? results from a multicenter study. J Am Coll Cardiol. (2012) 60:531–8. 10.1016/j.jacc.2012.04.032 22858289

[13] Hildick-Smith D Landmesser U Camm AJ Diener HC Paul V Schmidt B. Left atrial appendage occlusion with the Amplatzer™ Amulet™ device: full results of the prospective global observational study. Eur Heart J. (2020) 41:2894–901. 10.1093/eurheartj/ehaa169 32243499 PMC7421773

[14] Gaudino M Benedetto U Bakaeen F Rahouma M Tam DY Abouarab A. Off- versus on-pump coronary surgery and the effect of follow-up length and surgeons' experience: a meta-analysis. J Am Heart Assoc. (2018) 7:e010034. 10.1161/JAHA.118.010034 30373421 PMC6404195

[15] Beaver TM Hedna VS Khanna AY Miles WM Price CC Schmalfuss IM. Thoracoscopic ablation with appendage ligation versus medical therapy for stroke prevention: a proof-of-concept randomized trial. Innovations. (2016) 11:99–105. 10.1097/imi.0000000000000226 26914668 PMC6545892

[16] Beigel R Wunderlich NC Ho SY Arsanjani R Siegel RJ. The left atrial appendage: anatomy, function, and noninvasive evaluation. JACC Cardiovasc Imaging. (2014) 7:1251–65. 10.1016/j.jcmg.2014.08.009 25496544

[17] Lee R Jivan A Kruse J McGee EC Malaisrie SC Bernstein R. Late neurologic events after surgery for atrial fibrillation: rare but relevant. Ann Thorac Surg. (2013) 95:126–31. 10.1016/j.athoracsur.2012.08.048 23063202

[18] Hindricks G Potpara T Dagres N Arbelo E Bax JJ Blomström-Lundqvist C. 2020 ESC Guidelines for the diagnosis and management of atrial fibrillation developed in collaboration with the European association for cardio-thoracic surgery (EACTS): the task force for the diagnosis and management of atrial fibrillation of the European society of cardiology (ESC) developed with the special contribution of the European heart rhythm association (EHRA) of the ESC. Eur Heart J. (2020) 42:373–498. 10.1093/eurheartj/ehab648 32860505

